# Oral antivirals for COVID-19 among patients with cancer

**DOI:** 10.21203/rs.3.rs-3876022/v1

**Published:** 2024-01-24

**Authors:** Dorra Guermazi, Panos Arvanitis, Kendra Vieira, Jeremy L. Warner, Dimitrios Farmakiotis

**Affiliations:** Brown University; The Warren Alpert Medical School of Brown University; The Warren Alpert Medical School of Brown University; Rhode Island Hospital; The Warren Alpert Medical School of Brown University

**Keywords:** COVID-19, nirmatrelvir/ritonavir, molnupiravir, immunocompromised, cancer

## Abstract

**Purpose:**

Immunocompromised individuals, such as those diagnosed with cancer, are at a significantly higher risk for severe illness and mortality when infected with SARS-CoV-2 (COVID-19) than the general population. Two oral antiviral treatments are approved for COVID-19: Paxlovid^^®^^ (nirmatrelvir/ritonavir) and Lagevrio^^®^^ (molnupiravir). There is a paucity of data regarding the benefit from these antivirals among immunocompromised patients with cancer, and recent studies have questioned their efficacy among vaccinated patients, even those with risk factors for severe COVID-19.

**Methods:**

We evaluated the efficacy and safety of nirmatrelvir/ritonavir and molnupiravir in preventing severe illness and death using our database of 457 patients with cancer and COVID-19 from Brown University-affiliated hospitals. 67 patients received nirmatrelvir/ritonavir or molnupiravir and were compared to 56 concurrent controls who received no antiviral treatment despite being eligible to receive it.

**Results:**

Administration of nirmatrelvir/ritonavir or molnupiravir was associated with improved survival and lower 90-day all-cause and COVID-19-attributed mortality (p<0.05) and with lower peak O2 requirements (ordinal odds ratio [OR] 1.52, 95% confidence interval [CI] 0.92–2.56).

**Conclusion:**

Acknowledging the small size of our sample as a limitation, we concluded that early antiviral treatment might be beneficial to immunocompromised individuals, particularly those with cancer, when infected with SARS-CoV-2. Larger-scale, well-stratified studies are needed in this patient population.

## Introduction

Patients with cancer represent a diverse group, most of whom are at an elevated risk of severe illness and mortality when infected with SARS-CoV-2 [1]. They often exhibit additional risk factors for severe COVID-19, including older age, increased number of comorbidities, immunosuppressive therapies, metastatic disease, and frequent healthcare interactions [1]. Moreover, individuals with cancer, particularly hematologic malignancies (HMs), tend to mount weaker immune responses to COVID-19 vaccines than those without cancer [2, 3].

Both nirmatrelvir/ritonavir (Paxlovid^®^) and molnupiravir (Lagevrio^®^) are FDA-approved for the treatment of COVID-19 based on the results of two randomized clinical trials (RCTs) from previous phases of the pandemic [4, 5], with the latter being under emergency use authorization. Additionally, their effectiveness was confirmed in large retrospective registries, which included small proportions of immunosuppressed patients [6, 7]. That being said, several groups [4, 5, 8–11] have studied the beneficial role of those antivirals among eligible immunocompromised outpatients only, with relatively mixed results. A post hoc analysis from the aforementioned RCT [5] showed that molnupiravir treatment of mild-to-moderate COVID-19 in non-hospitalized, unvaccinated, immunocompromised adults was safe, but the clinical benefit from its administration, although numerically substantial, did not reach statistical significance [8, 11]. Importantly, adverse effects due to drug–drug interactions (DDI) between medications that immunocompromised patients may already be taking, and nirmatrelvir/ritonavir may impact its tolerability among patients with cancer [12–14]. Moreover, recently completed RCTs of both drugs showed a lack of clinical benefit from their use among low-risk patients or even vaccinated high-risk patients in the current era of Omicron variants and widespread immunity to SARS-CoV-2 [15–18]

To our knowledge, no studies to date have specifically appraised the effectiveness of molnupiravir and nirmatrelvir/ritonavir in preventing hospitalization and mortality, exclusively among patients with solid or hematologic malignancies, using appropriate, concurrent controls. In this study, we conducted a retrospective analysis utilizing patient-level data from our comprehensive institutional registry. We aimed to compare clinical outcomes between outpatients with cancer and COVID-19 who took molnupiravir or nirmatrelvir/ritonavir and concurrent controls, that is, patients with cancer who were diagnosed with COVID-19 and did not receive any antiviral treatment, although they were eligible for it.

## Methods

### Study design

**Figure 1:**
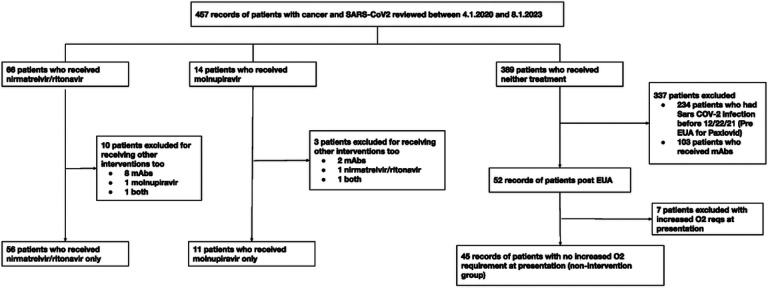
Patient selection. Flow diagram illustrating patient selection. *mAbs* Anti-spike monoclonal antibodies, *EUA* Emergency use authorization

We conducted a retrospective study at hospitals affiliated with Brown University. Our institutional database included all patients with active or historical malignancies diagnosed with COVID-19 between April 1, 2020, and August 1, 2023. Patients were excluded from the study if they met any of the following criteria: (1) they received anti-spike monoclonal antibodies (mAbs), the efficacy of which was shown in our previous study [19], (2) they had elevated oxygen requirements due to COVID-19 compared to their baseline needs, (3) they had COVID-19 before EUAs for molnupiravir or nirmatrelvir/ritonavir were issued (December 22, 2021), or (4) they were treated with both oral antivirals (Fig. 1). This study was approved by the Lifespan Institutional Review Board.

The outcomes of interest were (1) 90-day COVID-19-attributed (after exclusion of patients who died from other reasons) and all-cause mortality, (2) peak (worst) O_2_ requirements on a modified ordinal scale as follows: 0, outpatient only; 1, admitted to the hospital but without supplemental O_2_ requirement; 2, low-flow O_2_ requirement; 3, high-flow O_2_ requirement; 4, noninvasive mechanical ventilation (Bilevel Positive Airway Pressure (BiPAP), continuous positive airway pressure (CPAP)); and 5, invasive mechanical ventilation.

### Statistical analyses

Continuous variables are presented as medians with interquartile ranges (IQRs), while nominal and ordinal variables are shown as numbers with percentages. To compare differences between the two groups, we used the chi-square test or Fisher’s exact test as appropriate. We assessed 90-day survival using Kaplan–Meier curves, and differences between groups were tested with the log-rank test. To examine the relationship between molnupiravir administration, nirmatrelvir/ritonavir administration, and peak oxygen (O_2_) requirements, we used ordinal logistic regression analysis. We considered statistical significance at a two-tailed p value of 0.05.

## Results

### Baseline demographic and clinical characteristics

During the study period, 457 patients with cancer and SARS-CoV-2 infection were identified. Of the 389 (85%) patients who did not receive oral antivirals, 234 (51%) were excluded because they had COVID-19 prior to the availability of oral antivirals, 103 (23%) were excluded because they received mAbs, and 7 (2%) patients were further excluded due to increased O_2_ requirements at presentation. Thus, the control group consisted of 45 patients who (a) had not received molnupiravir, nirmatrelvir/ritonavir, or mAbs but were eligible for antiviral treatments since they (b) had SARS-CoV-2 infection after December 22, 2021 (post-EUA for nirmatrelvir/ritonavir), (c) did not have increased O_2_ requirements at presentation, and (d) were not admitted to the hospital for COVID-19. 56 patients received nirmatrelvir/ritonavir alone, and 11 patients received molnupiravir alone. Ten patients were excluded from the nirmatrelvir/ritonavir treatment group due to the administration of mAbs (8 such patients), molnupiravir (1 such patient), or both (1 such patient). Three patients were excluded from the molnupiravir treatment group due to the administration of mAbs (2 such patients), nirmatrelvir/ritonavir (1 such patient), or both (1 such patient).

The baseline demographic and clinical characteristics of these three groups were largely comparable (Table 1). 51% (57/112) of patients identified as male, although only 18% (2/11) of those in the molnupiravir group were male, and male gender was associated with higher mortality overall (Suppl. Table 1). 48% (54/112) of the patients were current or former smokers, and the most common comorbidity was hypertension (56%, 63/112). Most patients contracted COVID-19 in 2022 and 2023. There were no significant differences in vaccination status or the number of doses between groups.

Table 2 provides an overview of the distribution of cancer characteristics within all three groups. Most patients had solid tumors (70%, 78/112). Patients who received nirmatrelvir/ritonavir were more likely than their counterparts to have prostate cancer (controls: 2%, molnupiravir: 9%, nirmatrelvir/ritonavir: 14%). ECOG scores were not reported for 17 patients (8 controls, 8 who received nirmatrelvir/ritonavir and 1 who received molnupiravir). Patients who received nirmatrelvir/ritonavir were less likely than controls to have an ECOG score ≥2. We did not observe a clear association between ECOG scores and mortality (Suppl. Table 2). The most common anticancer treatment among patients who received nirmatrelvir/ritonavir was locoregional therapy (64%, 36/56), while cytotoxic therapy was most common for both the molnupiravir (91%, 10/11) and control (60%, 27/45) groups.

### Clinical outcomes

**Figure 2:**
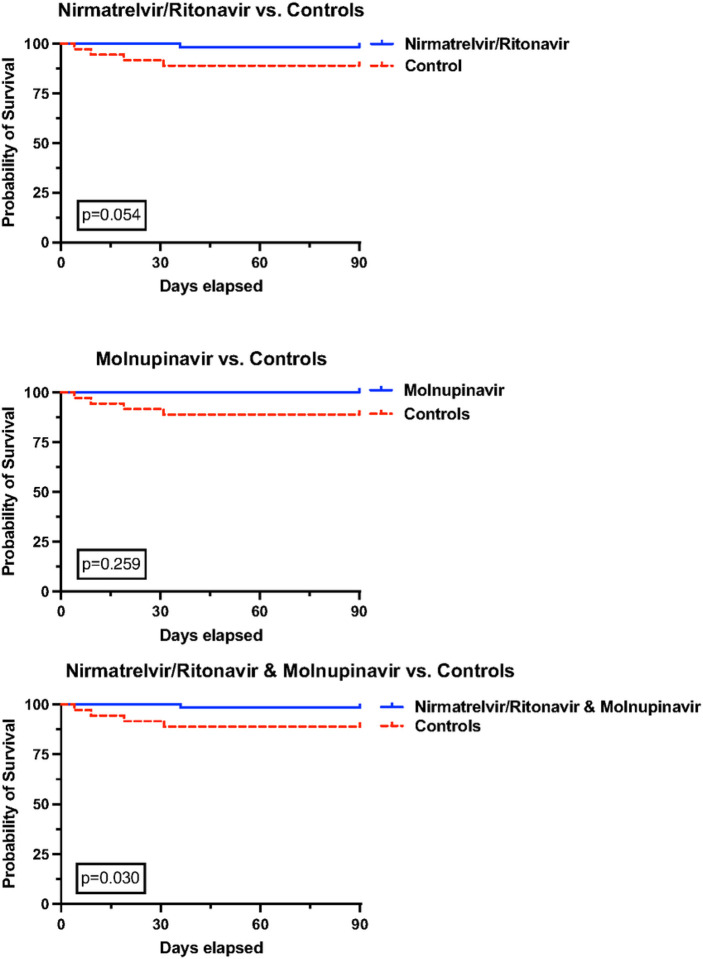
Kaplan–Meier survival curves. Kaplan–Meier 90-day COVID-19-attributed mortality curves for patients who received molnupiravir, nirmatrelvir/ritonavir and both for the treatment of SARS-CoV-2 infection and those who did not.

**Figure 3:**
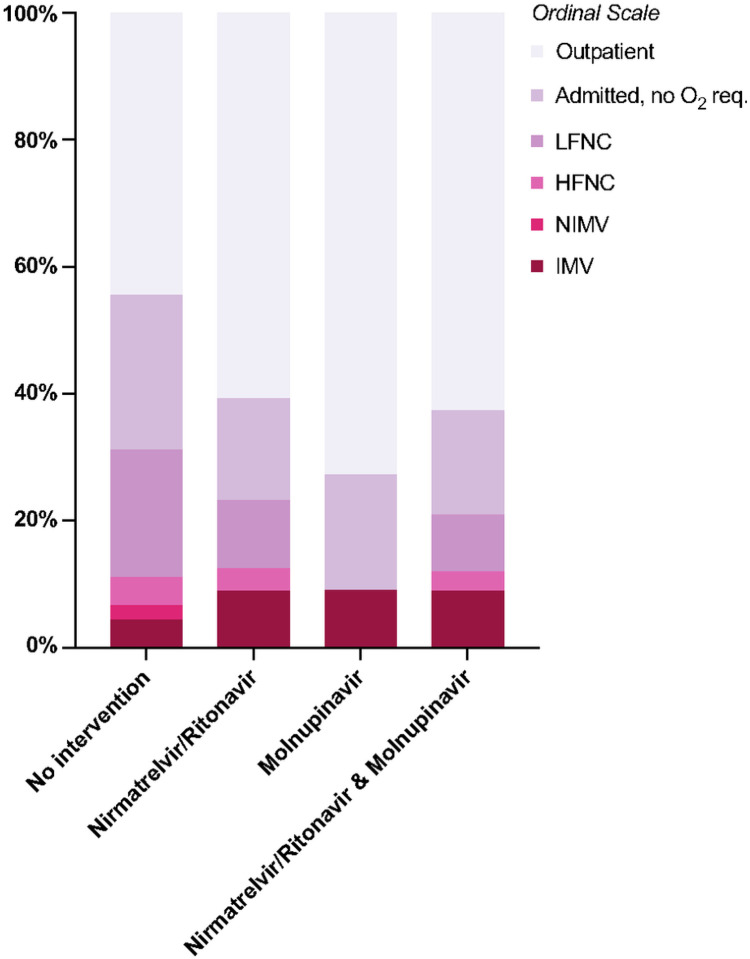
Peak O_2_ requirement ordinal scale value distribution by treatment status. *LFNC* low-flow nasal cannula, *HFNC* high-flow nasal cannula, *NIMV* noninvasive mechanical ventilation (BiPAP, CPAP), *IMV* invasive mechanical ventilation

Clinical outcomes are summarized in Table 3, and Kaplan–Meier survival curves are shown in Fig. 2. Of 56 patients who exclusively received nirmatrelvir/ritonavir, only 2 (3.6%) died, a 90-date mortality rate significantly lower than the 13 (28.9%) observed in the control group (p < 0.001) (Table 3). Similarly, of 11 patients treated with molnupiravir, none died (p = 0.042 compared to controls) (Table 3). When the patients who received either nirmatrelvir/ritonavir or molnupiravir were combined, they had a notably lower 90-day all-cause mortality rate (3.0% vs. 28.9%, p < 0.001) and COVID-19-attributed mortality rate (1.5% vs. 11.1%, p = 0.032) (Table 3). Additionally, the utilization of nirmatrelvir/ritonavir or molnupiravir was associated with improved survival, as demonstrated by Kaplan–Meier analyses in Fig. 2. Patients who did not receive nirmatrelvir/ritonavir or molnupiravir were 1.52 times more likely to have higher peak O_2_ requirements than patients who received nirmatrelvir/ritonavir or molnupiravir (ordinal OR = 1.52, 95% CI = 0.92–2.56) (Fig. 3).

## Discussion

To our knowledge, no studies have assessed the efficacy of nirmatrelvir/ritonavir and molnupiravir specifically among patients with cancer and COVID-19 to date. Although we cannot entirely rule out confounding from imbalances in baseline ECOG scores and male sex (for molnupiravir), we provide herein real-world evidence, using objective outcomes and appropriate controls, of potential clinical benefits from early administration of anti-SARS-CoV-2 oral antiviral medications in this vulnerable and growing patient population.

In addition to the seminal randomized controlled trial [4], two additional recent observational but large studies from China [20] and British Columbia [21] showed benefit from the administration of nirmatrelvir/ritonavir, especially among immunocompromised patients, in agreement with our results. However, vaccination coverage in the first two studies was remarkably low: unvaccinated [4] and 26.5% vaccinated [20], compared to our report (at least 3 doses of an mRNA vaccine in > 50% of patients in all groups, Table 1). Nevertheless, the study by Dormuth et al. [21] was performed in a highly vaccinated patient population (> 50% had 3 doses with approximately 30% 4 or more). That study showed incremental benefit from Paxlovid^®^ treatment in severely > moderately immunosuppressed individuals but no statistically significant benefit among non-immunosuppressed but otherwise high-risk patients with COVID-19. Deeply immunocompromised patients, especially those with hematologic malignancies, are at high risk for both severe COVID-19 [22] and poor response to vaccination [23, 24]. Our findings and those of the above studies indicate that such patients could benefit the most from nirmatrelvir/ritonavir and highlight the importance of risk stratification in the study of antiviral treatments among patients broadly considered “immunosuppressed”. Furthermore, the heterogeneity of patients referred to as “high-risk” for severe COVID-19 dictates caution in interpreting the recent results from randomized controlled trials showing no benefit from antiviral medications among vaccinated patients under that broad term [15, 17, 18].

It should be noted that DDIs between medications that oncologic patients often take and Paxlovid^®^ may significantly affect the risk-benefit ratio or even be prohibitive of its administration [12–14]. Nirmatrelvir, an antiviral protease inhibitor against SARS-CoV-2, is pharmacokinetically enhanced by ritonavir, a potent CYP3A4 inhibitor, to achieve therapeutic plasma concentrations [25]. This enhancement becomes critical when considering co-administration with tyrosine kinase inhibitors (TKIs), which are widely utilized in the targeted treatment of various malignancies, such as leukemia, non-small cell lung cancer (NSLC), and certain breast cancers, due to their primary metabolism via CYP3A4 [13]. Beyond TKIs, other commonly used chemotherapeutics, such as taxanes and vinca alkaloids, also share this metabolic pathway, heightening the risk of cumulative toxicity [26, 27]. The concomitant use of Paxlovid^^®^^ in patients with cancer, who might already exhibit elevated levels of chemotherapeutic agents due to the multifaceted impact of COVID-19 on drug metabolism and clearance, further complicates the therapeutic landscape [28]. These complexities underscore the need for a thorough evaluation of potential DDIs when using Paxlovid^®^, as well as careful monitoring and adjustment of chemotherapeutic dosing, to minimize the risk of enhanced toxicity while effectively managing both cancer and COVID-19.

The RCT data supporting the efficacy of Lagevrio^®^ among unvaccinated patients were weaker than those of Paxlovid^®^, and its EUA was supported by a marginal vote. A recent registry-based study claimed a significant benefit, almost similar to Paxlovid^®^, especially among elderly patients, even after adjustment for vaccination status and time from last vaccine dose [7]. Nevertheless, the PANORAMIC clinical trial [29], where > 96% of patients were fully vaccinated, showed no difference in clinical outcomes between molnupiravir and usual care alone, similar to the results of a recent systematic review and meta-analysis [16].

Despite hesitancy due to conflicting data, molnupiravir has gained some acceptance as an easily available, DDI-free oral treatment against COVID-19 in immunosuppressed patients taking multiple medications that could interact with ritonavir [4, 5, 8–11]. Again, the results are rather mixed: among 55 immunocompromised participants in a post hoc analysis from the MOVE-OUT trial, molnupiravir treatment demonstrated a noteworthy reduction in hospitalizations or deaths (8.3% vs. 22.6% for placebo) and a lower incidence of adverse events (25.0% vs. 45.2% for placebo) by day 29. However, none of these results were statistically significant [8, 11]. In another retrospective study of diverse immunocompromised US Veterans, > 50% of whom had received a vaccine booster, oral antiviral treatment was associated with a significant reduction in the composite outcome of hospitalization or death, largely driven by a decreased 30-day mortality rate. Of note, the investigators found similar magnitudes of benefit for molnupiravir and nirmatrelvir/ritonavir [30]. However, among lung transplant recipients, neither vaccination nor antiviral treatment with either remdesivir or molnupiravir had a significant effect on the odds of severe COVID-19, highlighting once again the importance of risk stratification within the “immunocompromised” patient population, with implications for decreased treatment benefits among the most immunosuppressed, especially those with concomitant structural lung disease [31]. To our knowledge, there are no published data on molnupiravir efficacy specific to the oncologic patient population. Although our sample size was too small to draw firm conclusions, no deaths occurred in the molnupiravir group. Our findings and the overall consensus that early antiviral treatment may be beneficial potentially support its use in selected patients when DDIs prohibit the administration of Paxlovid^®^.

Our study has several limitations. First, it was a single-center, retrospective study with a relatively small number of patients, although it was comparable to those of other similar reports [8–10, 20]. However, we used objective outcomes, which can be reliably abstracted from Electronic Medical Records (EMR). Utilizing concurrent controls and ensuring eligibility for treatment strengthens the study by preventing bias stemming from varying base mortality rates at different phases of the pandemic (an important caveat when using “historical controls” [32]) and by addressing potential confounding due to indication, respectively. Second, imbalances in ECOG scores and male sex could have influenced the outcome; however, the latter only applied to the small number of patients treated with molnupiravir. Furthermore, we analyzed COVID-19 attributable mortality to limit potential biases from cancer prognosis. Third, the number was too small to allow not only multivariable adjustments but also key subgroup analyses (e.g., among patients treated with rituximab or other anti-B-lymphocyte monoclonal antibodies), which should be the focus of future studies.

In conclusion, we found a signal for benefit from treatment of COVID-19 with an oral antiviral, especially nirmatrelvir/ritonavir, among patients with cancer. Importantly, our report and review of the literature highlight the need for larger samples and rigorous stratification of “high-risk” patients in observational studies and randomized controlled trials of anti-COVID-19 treatments.

## Figures and Tables

**Table 1:** Baseline characteristics

Parameter	Controls	Nirmatrelvir/ritonavir	Molnupiravir
Number of patients	45	56	11
Age (years) (median-IQR)	67 (54.5–79.5)	70 (61.6–78.4)	63 (57–69)
Male (%)	24 (53.3)	31 (55.4)	2 (18.2)
BMI (kg/m2) (median-IQR)	27.6 (22.6–32.6)	29.06 (25.2–33.0)	25.42 (19.9–30.9)
Race and ethnicity (%)			
Hispanic	2 (4.4)	6 (10.7)	2 (18.2)
Non-Hispanic Black	2 (4.4)	0 (0.0)	0 (0.0)
Non-Hispanic White	40 (88.9)	48 (85.7)	9 (81.8)
Other	1 (2.2)	2 (3.6)	0 (0.0)
Smoking status (%)			
Never	26 (57.8)	25 (44.6)	7 (63.6)
Current or former	19 (42.2)	31 (55.4)	4 (36.4)
Comorbid conditions (%)^a^			
Hypertension	24 (53.3)	33 (58.9)	6 (54.6)
Diabetes	22 (48.9)	20 (35.7)	3 (27.3)
Cardiac	16 (35.6)	25 (44.6)	4 (36.4)
CKD	8 (17.8)	5 (8.9)	2 (18.2)
Pulmonary	14 (31.1)	26 (46.4)	3 (27.3)
Year of contracting SARS-CoV-2			
2020	-	0 (0.0)	0 (0.0)
2021	3 (6.6)	1 (1.8)	0 (0.0)
2022	35 (77.8)	38 (67.9)	6 (54.6)
2023	7 (15.6)	17 (30.4)	5 (45.5)
mRNA vaccination status			
Unvaccinated	10 (22.2)	10 (17.9)	1 (9.1)
2 doses only	11 (24.4)	8 (14.3)	2 (18.2)
3 + doses	24 (53.3)	38 (67.9)	8 (72.7)
Received remdesivir			
Received remdesivir	16 (35.6)	12 (21.4)	0 (0)

Data are presented as number percentage (%) for categorical variables and median (interquartile range [IQR]) for continuous variables. All patients were coded as either female or male in the EMR; none were listed as intersex. Ethnicity and race data were taken from the hospital EMR and may not reflect patient self-identification.

*BMI* Body Mass Index, *IQR* interquartile range.

a Total will be greater than the total number of patients due to row overlap.

**Table 2:** Cancer characteristics

Parameter	Controls	Nirmatrelvir/ritonavir	Molnupiravir
Number of patients	45	56	11
Solid tumors	26 (57.8)	45 (80.4)	7 (63.6)
*Adrenal*	0 (0)	0 (0)	0 (0)
*Astrocytoma*	0 (0)	0 (0)	0 (0)
*Bladder*	1 (2.2)	0 (0)	0 (0)
*Breast*	6 (13.3)	10 (17.9)	2 (18.2)
*Cervical*	0 (0)	1 (1.8)	1 (9.1)
*Colon*	2 (4.4)	2 (3.6)	0 (0)
*Fallopian Tube*	0 (0)	0 (0)	0 (0)
*Gastric*	2 (4.4)	1 (1.8)	0 (0)
*Head and Neck*	1 (2.2)	0 (0)	0 (0)
*Liver Hemangioma*	3 (6.7)	1 (1.8)	0 (0)
*Lung*	2 (4.4)	4 (7.1)	0 (0)
*NSCLC*	1 (2.2)	3 (5.36)	0 (0)
*SCLC*	1 (2.2)	1 (1.8)	0 (0)
*Melanoma*	1 (2.2)	0 (0)	0 (0)
*Meningioma*	0 (0)	0 (0)	0 (0)
*Ovarian*	0 (0)	1 (1.8)	1 (9.1)
*Pancreatic*	2 (4.4)	7 (12.5)	0 (0)
*Prostate*	1 (2.2)	8 (14.3)	1 (9.1)
*Renal cell carcinoma*	1 (2.2)	2 (3.6)	0 (0)
*Testicular*	2 (4.4)	0 (0)	0 (0)
*Thyroid*	1 (2.2)	0 (0)	0 (0)
*Hematologic malignancy*	1 (2.2)	7 (12.5)	2 (18.2)
*Acute lymphoblastic leukemia*	1 (2.2)	0 (0)	0 (0)
*Acute myeloid leukemia*	1 (2.2)	0 (0)	0 (0)
*AL Amyloidosis*	0 (0)	0 (0)	0 (0)
*Chronic lymphocytic leukemia*	0 (0)	0 (0)	0 (0)
*Chronic myeloid leukemia*	1 (2.2)	1 (1.8)	0 (0)
*Hairy cell leukemia*	0 (0)	0 (0)	0 (0)
*Hemophagocytic Lymphohistiocytosis*	0 (0)	0 (0)	0 (0)
*Multiple Myeloma*	1 (2.2)	1 (1.8)	1 (9.1)
*Non-Hodgkin Lymphoma*	1 (2.2)	3 (5.4)	2 (18.2)
ECOG performance status (%)			
0	8 (17.8)	17 (30.4)	1 (9.1)
1	12 (26.7)	23 (41.1)	8 (72.7)
≥2	17 (37.8)	8 (14.3)	1 (9.1)
Unknown	8 (17.8)	8 (14.3)	1 (9.1)
Anticancer therapy modality (%)^a^			
None	3 (6.7)	1 (1.8)	0 (0)
Cytotoxic	27 (60.0)	32 (57.2)	10 (90.9)
Locoregional (surgery and/or radiation)	22 (48.9)	36 (64.3)	7 (63.6)
Immunotherapy	14 (31.1)	17 (30.4)	2 (18.2)
Targeted	10 (22.2)	8 (14.3)	2 (18.2)
Endocrine	6 (13.3)	11 (19.6)	2 (18.2)
Antimetabolite	10 (22.2)	10 (17.9)	1 (9.1)

Data are presented throughout as numbers (percentages).

*ECOG* Eastern cooperative oncology group, *NSCLC* Non-small cell lung cancer, *SCLC* small cell lung cancer.

a Total will be greater than the total number of patients due to row overlap.

**Table 3:** Clinical outcomes

	Nirmatrelvir/Ritonavir	Controls	p value
PRIMARY OUTCOME			
No. of patients	55	36	0.057
Covid related death	1 (1.8)	4 (11.1)	
SECONDARY OUTCOME			
No. of patients	56	45	**<0.001**
All-cause mortality	2 (3.6)	13 (28.9)	
	Molnupiravir	Controls	p value
PRIMARY OUTCOME			
No. of patients	11	36	0.248
Covid related death	0 (0.0)	4 (11.1)	
SECONDARY OUTCOME			
No. of patients	11	45	**0.042**
All-cause mortality	0 (0.0)	13 (28.9)	
	Nirmatrelvir/Ritonavir & Molnupiravir	Controls	p value
PRIMARY OUTCOME			
No. of patients	66	36	**0.032**
Covid related death	1 (1.5)	4 (11.1)	
SECONDARY OUTCOME			
No. of patients	67	45	**<0.001**
All-cause mortality	2 (3.0)	13 (28.9)	

Statistically significant *p* values (*p* < 0.05) are highlighted in bold.
